# Synergies and fragmentation in country level policy and program agenda setting, formulation and implementation for Global Health agendas: a case study of health security, universal health coverage, and health promotion in Ghana and Sierra Leone

**DOI:** 10.1186/s12913-021-06500-6

**Published:** 2021-05-20

**Authors:** Irene Akua Agyepong, Fredline A. O. M’Cormack-Hale, Hannah Brown Amoakoh, Abigail N. C. Derkyi-Kwarteng, Theresa Ethel Darkwa, Wallace Odiko-Ollennu

**Affiliations:** 1 Ghana College of Physicians and Surgeons, 54 Independence Avenue, Accra / Dodowa Health Research Center, P.O. Box DD1, Dodowa Accra, Ghana; 2 School of Diplomacy and International Relations, 109 McQuaid Hall, Seton Hall University, 400 South Orange Avenue, South Orange, NJ 07079 USA; 3 Noguchi memorial Institute for Medical Research, University of Ghana, P.O. Box LG 581, Accra, Ghana; 4 University Medical Center, Utrecht University, P.O Box 855003508, GA Utrecht, The Netherlands; 5 Ghana Health Service / Ghana College of Physicians and Surgeons, Accra, Ghana; 6 Public Health Division, 37 Military Hospital, Neghelli Barracks, Cantonments, Accra, Ghana; 7 Non-Communicable Diseases Control Program, Ghana Health Service, P.O.Box KB 493, Korlebu Accra, Ghana

**Keywords:** Synergies, Fragmentation, Agency, Context, Power, Global agendas, Low and middle income countries (LMIC), Universal health coverage (UHC), Health security (HS), Health promotion (HP)

## Abstract

**Background:**

Global health agendas have in common the goal of contributing to population health outcome improvement. In theory therefore, whenever possible, country level policy and program agenda setting, formulation and implementation towards their attainment should be synergistic such that efforts towards one agenda promote efforts towards the other agendas. Observation suggests that this is not what happens in practice. Potential synergies are often unrealized and fragmentation is not uncommon. In this paper we present findings from an exploration of how and why synergies and fragmentation occur in country level policy and program agenda setting, formulation and implementation for the global health agendas of Universal Health Coverage (UHC), Health Security (HS) and Health Promotion (HP) in Ghana and Sierra Leone. Our study design was a two country case study. Data collection involved document reviews and Key Informant interviews with national and sub-national level decision makers in both countries between July and December 2019. Additionally, in Ghana a stakeholder workshop in December 2019 was used to validate the draft analysis and conclusions.

**Results:**

National and global context, country health systems leadership and structure including resources were drivers of synergies and fragmentation. How global as well as country level actors mobilized power and exercised agency in policy and program agenda setting and implementation processes within country were also important drivers.

**Conclusions:**

There is potential in both countries to pull towards synergies and push against fragmentation in agenda setting, formulation and implementation of global health agendas despite the resource and other structural constraints. It however requires political and bureaucratic prioritization of synergies, as well as skilled leadership. It also requires considerable mobilization of country level actor exercise of agency to counter sometimes daunting contextual, systems and structural constraints.

**Supplementary Information:**

The online version contains supplementary material available at 10.1186/s12913-021-06500-6.

## Background

## Introduction

UN Member states including most LMIC have committed to the global health agendas in the SDG which include universal health coverage (UHC), health security (HS) and health promotion (HP). Given there is sometimes variation in meaning when the terminology UHC, HS and HP are used, to avoid ambiguity we make explicit that in this paper we use UHC, HS and HP in the meanings adopted in the ongoing work of the Lancet Commission on Synergies. By UHC we refer to the most important activities to ensure *access for everyone to quality essential health-services, including medicines and vaccines, and to financial risk protection (linked to payment for services).* By HS we refer to the most important activities required for *reduced vulnerability of people to rapidly spreading risks to health, particularly those with great potential to cross international borders.* By HP we refer to the most important *activities to enable people to increase control over the determinants of their health and to change social, environmental and economic conditions for better health* i.e. *enabling healthy lives.*

Global health agendas, have the common end goal of contributing to population health improvement; and policies and programs to attain them are formulated and implemented within the same health system at country level. In theory therefore, policies and programs should be formulated and implemented in ways that ensure synergy. By synergy, we refer to the combined ability to achieve the desired goal of policies, programs and interventions when they work together that is greater than what can be achieved by each working separately. Health Systems are complex adaptive systems; and within health systems, “problems” are part of a wider interconnected set of linkages, relationships, interactions and behaviours [1]. To intervene effectively in such systems with the goal of population health outcome improvement requires understanding and a focus on maximizing the effectiveness of the whole rather than of selected components at the expense of the whole. By fragmentation we refer to separating to various degrees policies, programs and interventions to maximize the achievement of goals related to selected components while ignoring to various degrees the whole of which they are a part.

In theory, given chronic resource constraints, low and middle income countries stand to gain by prioritizing synergistic approaches to global health agendas whenever possible. Observation suggests that this is not necessarily what happens in practice. Different stakeholders may pursue their selected agendas without reference to other agendas within the same system [2, 3]. An important question is whether there is an evidence base that proves that synergies are required and useful and fragmentation is not. Much of the still limited literature related to this question uses the language of integration rather than synergies. Integration refers to coordinating or blending into a unified whole. Integration and synergies can be considered as part of a spectrum of systems thinking approaches, from tightly bound into a whole (integration) to more loosely knit intersections (synergies). A systematic review of the evidence on integration of targeted health interventions versus integrating health systems and the interventions [4] suggests a highly heterogenous picture both for the nature and extent of integration and a limited evidence base for integration versus targeted programing and vice versa. “Success” is measured with different indicators such as efficiency, effectiveness, and equity further complicating the ability to draw clear lessons as to whether integration is “good” or “bad”.

In the context of a still limited evidence base despite decades of debate, and continuing strong opinions, about a complex phenomenon like fragmentation and synergies; that is influenced by multiple variables and varying contexts making it difficult to use statistically generalizable approaches to evaluation; we do not focus on trying to conclusive prove or disprove synergies “good” or “bad” as a starting point for our work. We rather start from a normative position based on our conclusions from studying the literature, listening to varying opinions and experience from working in the health system in Ghana and Sierra Leone over many years, that whenever possible synergies are more desirable than fragmentation and should be actively pursued. From that normative position we ask and explore questions about how and why synergies and fragmentation occur (or not) in policy and program agenda setting, formulation and implementation for global health agendas; specifically focusing on UHC, HS and HP in Ghana and Sierra Leone. We do this to generate evidence to inform global and country level decision making and implementation of global health agendas with the ultimate goal of population health improvement.

### Theoretical concepts

We drew on theoretical concepts of the policy process, actor agency and power, health systems and context from the policy analysis and social science literature to inform the themes we explored in our data collection. To conceptualize the policy process, we drew on a common adaptation of Lasswell’s [5] modeling of the policy process into four stages of agenda setting, formulation, implementation and evaluation. Despite valid critiques [6] that this model is a somewhat unrealistic linear conceptualization of an iterative and unpredictable process; it retains value in organizing thinking and analysis. By the agenda we refer to: *“the list of subjects or problems to which government officials, and people outside the government closely associated with these officials are paying serious attention at any time”* [7]. Formulation describes the cluster of complex decision-making processes between agenda setting and implementation [8]; and implementation to the execution of decisions to make a program happen [9, 10].

Actor agency in sociology refers to the capacity of people to act independently and make their own free choices [11, 12]; while power is the capacity to exercise that choice and effect (or affect) and influence decisions and actions. Levers of power include the *control* of a resource, a technical skill, or a body of knowledge; *authority* by virtue of one’s legal and structural position; and *access* to those who can rely on the other sources of power. Sources of power can be visible e.g. vested in formal rules, structures, and decision-making procedures or invisible e.g. shaping how people think about issues, give meaning and decide what is acceptable (or not); or *hidden* with power used to control who gets to participate in decision making and what items make it to the decision-making agenda [13, 14]. We theorized that country as well as global actors have and exercise agency and varying levers of power to to influence synergies and fragmentation.

Health systems are the primary arena in health policy and program agenda setting formulation and implementation occur. We drew on the WHO definition of a health system as consisting of: “*… all organizations, people and actions whose primary intent is to promote, restore or maintain health*” [15]; and the several frameworks for describing and analyzing health systems in the literature; especially frameworks with a social constructivist approach that put people at the center of health systems [15–21]. Context refers to the circumstances that form the setting for a phenomenon. We theorized that context can push policy processes towards synergies or pull them away from synergies. We categorized context into environmental, situational, structural, and cultural [22]. Environmental or global context describes context outside the country itself such as the international political environment, agreements, obligations and pressures. Situational context refers to transient, impermanent events that have an impact on decision making such as wars, communal conflict, terrorism, economic cycles, epidemics and pandemics such as Ebola and COVID-19, droughts, floods, oil spills and earthquakes. Structural context comprises the more permanent features of a systems such as the economic base, political institutions and demographic structure. Cultural context refers to norms and values, formal and informal political culture norms and values, traditional social values, institutions and arrangements such as marriage, the family, religion etc.

## Methods

### Aim

The aim of this study was to explore how and why synergies occur (or not) between the global health agendas of UHC, HS and HP in Ghana and Sierra Leone.

### Study design

The study design was a multiple (two) country qualitative exploratory case study in Ghana and Sierra Leone. The case was defined as: *“UHC, HS and HP country level policy and program agenda setting, formulation and implementation over the period 1992 - 2019”* We chose a case study approach to enable us to explore in depth a complex phenomenon where it is difficult to separate the phenomenon from the contexts in which it occurs. We purposively selected Ghana and Sierra Leone for the case studies to study the issues in contrasting socio-economic and developmental country contexts.

### The case study countries

Table 1 presents selected contextual indicators for the two countries. Structurally, the period covered by this study was one of political stability and economic growth in Ghana during which it moved from a low-income to a lower middle-income country. The December 1992 elections ushered a change from a preceding unstable period of attempts to establish multi-party democracy cut short by military coups to the stable multi-party democratic governance of the fourth republic. Since 1992, there have been back to back elections every 4 y with peaceful transitions of power between two major political parties – the National Democratic Congress (NDC) and the New Patriotic Party (NPP). The same period was one of political and economic turbulence for Sierra Leone with two major situational crisis. The first was a devastating 11 years of civil war between 1991 and 2002. The second was the 2014–2016 West African Ebola outbreak, of which it was an epicenter. Politically, Sierra Leone is a multi-party democracy with two main political parties, that have alternated power since independence in 1961: the All People’s Congress (APC) and the Sierra Leone People’s Party (SLPP) with a series of coups and countercoups in-between. Entrenched poverty and excesses such as patrimonialism, and corruption have been blamed for the 1991–2002 civil war. Since the end of the Civil war in 2002, there have been peaceful multi-party democratic elections with back to back transfers of power between the two dominant political parties.

**Table 1:** Selected Contextual indicators for Ghana and Sierra Leone (Data sources [23–27])

	Ghana	Sierra Leone
Population	30,417,856	7,813,215
GDP per capita	$ 2202	$ 528
2019 Human Development Index (HDI)	•0.596 •Medium human development category •142 out of 189 countries and territories.	•0.438 •Low human development category •181 out of 187 countries and territories.
Poverty Indicators	•Overall poverty head count ratio 23% •Population in extreme poverty 8%.	•Overall poverty headcount ratio 57%, •Population in extreme poverty 11%.
Life expectancy	64 years	54 years
Fragile State Index	68	89

### Sampling, data collection methods and tools and analysis

In both countries data collection involved an initial desk review of grey and published literature. It was followed by key informant interviews to fill in the gaps in the evidence from the desk review. To validate our initial analysis and conclusions we held a stakeholder validation meeting in Ghana in December 2019.

#### Desk review

In both countries, additional to a google search; the research team searched the websites, institutional libraries and archives of the Ministries of Health; and of health sector agencies such as the Ghana Health Service, Non State providers e.g. Religious Health Associations; development partners and Civil Society Organizations (CSO) for policy and program documents. In Sierra Leone, the team also reviewed Service Level Agreements (SLA) that were part of documents stored by the Integrated Health Planning Administration Unit (IHPAU). Our search was non-exhaustive and iterative in keeping with our flexible qualitative methodology. We read and analyzed documents as we went along; and stopped when we had reached saturation in that no new information was emerging from continued searching. Key words and phrases we used to search for documents online or scan titles and abstracts of hard copy documents included ‘synergies’, ‘fragmentation’, ‘UHC’, ‘HP’, ‘HS’, ‘policy’, ‘agenda’, ‘program’, ‘implementation’, ‘context’, ‘Ghana’ and ‘Sierra Leone’.

#### Key informant (KI) indepth interviews

The Key Informant (KI) interview topic guide design was informed by the analysis of the desk review findings. The design involved critical review by all members of the research team. The topic guide was not pretested after this because of the flexible qualitative exploratory research design we used. The emphasis was a topic guide to focus exploratory discussion to gain in-depth understanding rather than a fixed set of questions. The Research team analyzed the information from the interviews as they emerged rather than at the end of all data collection. This flexible approach was necessary because our primary interest was to qualitatively explore and be able respond to emerging information and probe further if indicated; rather than prove any pre-existing hypothesis. The strength of this approach is the deep qualitative insights provided. The weakness is that no attempts at statistical generalizability can be made. The process can however be replicated and the topic guide itself is made available as a supplementary file to this paper.

In both countries selection of KI was purposive. In Ghana KI were immediate past and present senior mainly national level decision makers from Government (MOH and its agencies), as well as Non-Government /non-State institutions and donor agencies active in the health sector. KI were initially identified from the research team’s knowledge of the health sector and from the desk review. A snowballing approach was used to follow suggestions from people already interviewed as to who else to interview. A total of 27 KI interviews were conducted. Twenty (20) KI were still in active service and seven (7) had retired. Fifteen (15) were or had been public sector employees (MOH and its agencies) at the level of director or deputy director. Of these thirteen (13) were working or had worked at national level, one (1) was at regional and one (1) at district level. Of the remaining twelve (12) from the non Government sector, ten (10) were employees of external Development Partners (DP) active in Ghana and two (2) were from Ghanaian non-state agencies. Twenty two (22) were male and four (5) were female. In Sierra Leone, sixteen (16) KI were from the public sector (MOH) and two (2) from the donor and NGO community making a total of eighteen (18) KI. There were 16 males and 2 females. A first set of interviews was held with KI within the MOH at the district level based on previous work in the health sector and knowledge of who the key stakeholders were. Using a snowballing approach, the initial KI helped to identify other KI. The respondents included district medical officers, chief and medical superintendents, councilors and senior nurses across most of the districts in Sierra Leone. We wanted to maintain a more balanced male: female ratio of respondents but it was not possible because senior decision making levels in both countries had more men than women represented. All KI interviews were conducted between July and December 2019.

#### Stakeholder validation meeting

A stakeholder validation meeting was held in December 2019 in Accra with participants in the Ghana KI interviews, as well as people we had wanted to interview but who had not been available at the time of the interviews; to present our preliminary analysis and conclusions for critical feedback and a reality check on their validity. To help assure open transparent discussion and independent points of view, the meeting observed the Chatham house rule [28].

### Data processing and analysis

Apart from one interview in Ghana where the KI declined to be recorded and the research team had to rely on interviewer notes only; all KI interviews were tape recorded with informed consent. The tapes were transcribed by research assistants who did not participate in the interviews and did not know the respondents. Data analysis involved manual text analysis of transcripts for themes, commonalities and contrasts related to the theoretical concepts as well as new emerging themes. In Ghana two members of the research team (ADK and HBA) conducted the interviews with TED and WOO assisting. Three members (IAA, ADK and HBA) independently read through the transcripts and identified themes and then discussed and reconciled their conclusions as part of quality assurance. Analysis was done on an ongoing basis alongside the interviewing and transcribing. Where there was any disagreement the transcripts were revisited and if indicated the area was probed in further depth in subsequent interviews. In Sierra Leone three data collectors were hired to collect the data. Prior to embarking on data collection, they were trained by a member of the research team (FMH), on research objectives, methodology, informed consent and the questions. FMH and a research assistant independently read through the transcripts, identified themes and discussed conclusions as part of quality assurance.

### Ethical considerations/ethical issues of concern

Ethics approval was obtained from the Ghana Health Service Ethics Review Committee (GHS-ERC 007/03/19. Approval date 9th July 2019). All interviews were conducted with Informed consent. All transcripts were stored with utmost confidentiality in a password protected database accessible only to the researchers and used solely for answering questions related to this study. Respondents are identified by codes rather than by names. In this paper, to preserve anonymity, we label respondents by their institution as: “MOH /State”; “Non-State Partner” and “Development Partner”.

## Results

*‘I think that it’s unfortunate ... that there's not been a conscious effort to link them up … .’ (MOH /State Ghana).*

Synergies and fragmentation were perceived to be occurring between all three agendas, across all levels of the health system as well as in the different stages of the policy cycle in both countries. Synergies were generally perceived as beneficial and fragmentation as undesirable. However, some respondents felt fragmentation was sometimes needed to quickly achieve specific targets. KI felt that international agendas sometimes offered opportunities for synergy e.g. SDG and Primary Health Care (PHC) and sometimes for fragmentation e.g. Global Fund. Within the countries, there was some discrepancy between narrative in favor of synergies versus practice that did not promote synergies. For example, in Sierra Leone, although development plans appeared to focus synergistically on broad development outcomes and incorporate at least two or all three agendas, implementation was sometimes single issue focused.

**Fig. 1:**
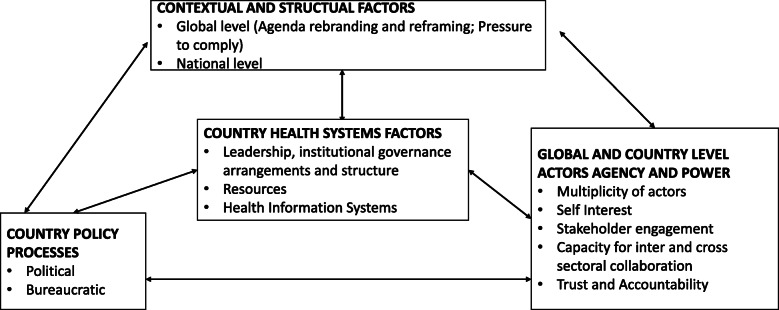
Why do synergies and fragmentation occur (or not) in country level policy and program agenda setting, formulation and implementation for HS, UHC and HP in Ghana and Sierra Leone

We summarize findings as to how and why synergies occur (or not) in Fig. 1. The factors in Fig. 1 act interactively and iteratively rather than linearly or individually. We use two way arrows to illustrate this.

### Country policy processes

The national political arena was the highest level of agenda setting and the two main political parties in both countries consistently made high level policy agenda statements on health in their manifestos. These statements reflected aspects of all three agendas but especially UHC and HP without necessarily using the terminology of UHC, HS or HP.

In Ghana, the NPP election manifesto for the December 2000 and 2004 elections which they won reflected these agendas in statements such as: **“***Recognising that the major causes of diseases and premature death in Ghana result from deficiencies or defects in our social, cultural and economic environment, the NPP government will vigorously pursue the removal of these handicaps as prescribed in other parts of this Manifesto. Its health policy will be devoted to health promotion and disease prevention as a priority. In addition, the care of the sick will be pursued on an equitable basis so that the needs of the sick are addressed according to their circumstances rather than their ability to pay..*.” [29] Similarly, the NDC manifesto statements for health for the 2008 elections which they won reflected all three agendas with phrases such as: “*Promoting health, preventing illness, providing cure, … extend access to health services … Provide clean and portable water and increase sanitation … .. Extend decent, affordable houses for a healthier living environment … ..Continue high level support for HIV/AIDS and TB initiatives”* [30]. Their manifesto for the 2012 election focused similarly on access, equity, affordability and inclusion for communicable and non communicable diseases. In the 2016 election manifesto of the NPP the terminology of HS and cross border communicable disease control distinctly emerges for the first time, probably reflecting the West African experience of the 2014–2016 Ebola outbreak. Additional to previous language such as: *‘ … Right to Health through health promotion, disease prevention.. improve access … ,*’ the 2016 manifesto states: ‘*The NPP seeks to position Ghana to address comprehensively local, national and emerging global health concerns* e.g. *Zika and Ebola viruses … ..”* [31]

In Sierra Leone, UHC related agendas were reflected in framing and statements around expanding access as well as affordability of health services. For example in their 2018 election manifesto the SLPP indicates an agenda to: *‘… transform the under-resourced, ill-equipped, dysfunctional and inadequate health infrastructure and healthcare delivery system to make it high quality, efficient, reliable, cost-effective, affordable and sustainable capable of responding to epidemics such as cholera, Ebola Virus Disease. …*. *increasing access of the population (particularly mothers, children and the elderly) to quality health services in an equitable and efficient manner.”* [32] *The* ACP also indicate that in their list of priorities is: “*Expanding access to and improving the quality of services in our health, education, water, energy and justice sectors”* [33]. Health promotion was reflected as an intent to address factors that contribute to poor health outcomes such as water and sanitation and nutrition. Before the 2014 Ebola outbreak the language in relation to diseases with epidemic potential predominantly reflected framing related to preventing and controlling communicable diseases rather than dealing with cross border communicable disease spread threats.

The broad framing of political agendas for health meant that senior civil servants immediately below the political leadership were important in shaping the detail of policy formulation and thus held potential to ensure synergies or fragmentation.

*‘ … basically, we influenced a lot of things that happened … .. In health, it is the technocrats that are responsible for policies … The difficulty they are having now in the ministry is that the technical people they have there are very young, they lack the experience’ (MOH /State Ghana).*

The high political leadership turnover in both countries made the role of these senior civil servants even more important.

*‘ … for the seven years that I was Director of Health Services, I served under four ministers. So, their lifespan was about one and half years. So, they’re just passing by … (MOH / State Ghana)*

### Contextual and structural factors

#### Global agenda rebranding and reframing

*‘ … ..there is a concept UHC, so we’re implementing UHC, there is a concept MDG, we're implementing MDG and MDG comes in components, so we’re implementing maternal health … .’ (Non State Partner Ghana).*

Several KI felt that the concept of the 3 agendas was not new. Rather a rebranding and reframing that occurs periodically in global health was occurring once more. This was itself a source of fragmentation. It sometimes caused some distraction at country level from consistently pursuing long term goals. New programs to do the same thing were set up rather than continuing and reinforcing already existing programs. For example, though in health sector policy and program documents pre-2015; the terminology of UHC and HS were not necessarily used the principles were felt to be inherent in the meaning of the text. In Sierra Leone, it involved efforts to improve the quality and efficiency of health care making it more affordable, and available to more people, with particular emphasis on vulnerable populations of mothers, and children under 5. In Ghana, the National Health Insurance scheme (NHIS) legislation was passed in 2003 when the terminology of UHC was not in use. However, the policy and program were effectively a UHC effort. In both countries Health Security was largely reflected in measures undertaken to enhance the ability of the country to protect citizens from infectious diseases, through prevention, early identification, response and containment. Following the 2014–2016 West African Ebola outbreak the terminology of “Health Security” began to increasingly appear. HP was conceptualized through the period studied in both countries in terms of promoting health by influencing social determinants that can lead to better quality of life, including health education, nutrition, environmental protection as well as sanitation and hygiene; community engagement and social mobilization.

#### Global pressure to comply

" …. *As a State or as a Ministry, you are bound to comply with international benchmarks. Otherwise you will stand to lose, and you know what will befall you and your government." (MOH /State Sierra Leone)*

Like most other LMIC, Sierra Leone and Ghana are members of global international bodies and signatory to a host of international declarations and policies. Governments try to align to these international policy commitments regardless of whether the agenda supports synergies or fragmentation. Of more concern is that failure to align could have negative consequences such as loss of desperately needed Development Partner (DP) funds. For example, considerable DP resources have been poured into addressing infant and child mortality, in Sierra Leone. It was felt the focus could be purely due to Sierra Leone having one of the highest maternal and child mortality rates in the world [34]. However, it also appeared to be due in part to the irresistible lure of the resources that follow global health priorities. Similarly, the Ebola outbreak commanded significant global attention and resources. Ebola was couched as a security threat: global powers were concerned about the threat the disease posed to their own citizens, as well as to international stability. To quote the then US Secretary of Defense: “*such events threaten not only the health of our citizens but also geopolitical stability ... When states are unable to provide basic services for their citizens, dangerous regional and global security consequences result*.” [35]. In September 2014, the UN Security Council convened over a health issue for the first time, calling the outbreak a “*threat to international peace and security*” [36] These concerns translated into increased resources during and after the Ebola outbreak. The greater prioritization of HS, is partly evidenced in the number of laws passed in Sierra Leone that reference HS. The speed with which policies were developed meant that they were not necessarily cohesive or synergized with pre-existing policies.

#### National Contextual drivers

The severity of the 2014 Ebola outbreak in Sierra Leone, and the inability of the government to successfully contain it for so long was seen by some as a result of the health system fragmentation in the immediate post Civil war reconstruction period as well as the mistrust between citizens and the state engendered by years of neglect and policies that reinforced inequity and marginalization [37]. Post war efforts in rebuilding were largely confined to infrastructure, prioritizing this over investments in staffing. There was also a DP preference for funding selected diseases like malaria, HIV/AIDs and TB [38].

However, the 2014 Ebola outbreak in Sierra Leone helped to raise attention to fragmentation, despite also being a cause of fragmentation during and after its eventual successful containment. During the outbreak, there were many calls to re-think development work in the health sector [39] and focus on health systems strengthening rather than vertical projects. A good number of respondents saw the Ebola outbreak as well as the post war context all as incentives, or imperatives to help push a synergistic agenda and to move away from fragmentation. Some felt that some of this opportunity was realized. e.g.:

*“... The post Ebola security agenda has helped to improve synergies in the national health policy making as there is strong coordination between MOHS and Donor partners in terms of health service delivery …. . " (MOH / State Sierra Leone).*

However, despite these positive expressions and testimonials expressed by some government (State) respondents, others felt that in practice, what was strengthened was the State’s ability to address HS. It was less clear, the extent to which this translated into greater synergies with other agendas. Thus it was felt that the aftermath of Ebola was a: “*mixed blessing …* o*n the one hand, the resources made available in the wake of Ebola have brought new and heretofore unseen attention and energy to areas such as human resources for health (HRH), medical products and information systems .... On the other hand, as the health sector has become more diverse, it has also become more fragmented ….* “ [40].

### Global and country level actors, agency and power

#### Multiplicity of actors

In both countries, there was a multiplicity of institutional actors in the health system. Mechanisms for DP coordination in Sierra Leone were fragmented, with many different management meetings and structures both within and without the health sector and a lack of clarity regarding who or what body was responsible for what. For example, there was a health development partners forum, as well as a health NGO forum. Coordination bodies included a Health Sector Coordinating Committee (HSSC), a Health Sector Steering Group (HSSG) and technical working groups.

At the time of this study, the trend among DP in Ghana was donor transitions out of the health sector since the country had been reclassified from low income to lower middle income. The country had also reverted to the fragmentation that existed before the Sector Wider Approach (SWAp) of the 1990s. During the Ghana sector wide approach (SWAp) that started implementation in 1997 several DP put their funds into the donor pooled fund. In exchange they took part in agenda setting, policy formulation and developing comprehensive and synergistic health sector annual and medium term plans as well as their monitoring and evaluation. The Ghana SWAp gradually died out as actors in the sector changed; and with global ideology shifts, the DP involved in the SWAp moved to donor budget support in the ministry of finance. When donor budget support ended most DP reverted to selective program support.

Media and CSO were mentioned as part of the multiplicity of actors in the policy process. Though Academia was mentioned as a policy actor in agenda setting, formulation and implementation in both countries; there was a perception that the focus of academia was rather “different” and more on conducting research than engaging in the policy process.

#### Self interest

*‘ … … everything is done and then you are not seen even though you have contributed so much... So if I get my own resources, I get my own thing and I push for my own level; then that will enable me to achieve my own goals, , … but I will be implementing it in maybe competition with another same player within the same ministry or agency’ (Non State Partner Ghana).*

*‘it’s the way we’ve set up our system; that there are divisions responsible for certain things. So, then they’re, like protective of their turf …. So, they shove people off, and they do their mandate’(MOH/ State Ghana).*

Several KI observed that institutions and individuals are keen to protect their visibility and interests in the policy process. These concerns can over-ride any common good concerns about synergies. Actors are less likely to work synergistically if it reduces their visibility.

Self interest leading to fragmentation was also mentioned as occurring because of the jostling of professional groups for power and influence to promote the welfare of their particular group.

*‘ … what I’ve observed (about the position of the minister), anytime it’s a medical doctor, all other groups will fight him or her. If it’s a pharmacist, all other groups will fight because they think that, … If you put a pharmacist there, everything (is) reserved for pharmacists.. If you put a nurse or whatever … , he/she will promote (that professional group) …. ’ (MOH /State Ghana).*

Political machinations, the lack of institutional memory due to politically motivated appointments and reshuffles, the ownership of clinics by senior officers within the health administration, suggesting tensions between job performance and personal gain, has led some writers to suggest that fragmentation is Sierra Leone is actually a deliberate self interested policy [41].

#### Stakeholder engagement

*‘ … involvement in the policy discussion has narrowed to some few people, even within the ministry.... I think stakeholder involvement is very minimal, and I think that’s where the worry is....’. (MOH /State Ghana)*

Although there were various regular meetings for health sector actors in Ghana, KI observed that important actors were often missing during the discussions. This leads to a narrow scope of discussion, viewpoints and ideas as well as misunderstanding of roles and approaches to implementation that drives fragmentation. Related to these concerns about stakeholder engagement were concerns about perceptions of who matters in the policy process.

*‘ … who are the key or who should be, they are two things; … .others are left in the periphery by the view that it is policy … service providers are only to implement policy; which for me is absurd because policy doesn’t refer to only the formulation stage but a whole cycle of processes, and a policy without implementation is no policy’. (MOH / State Ghana)*

There appeared to be dominance of a top down model of “policy making” which several KI felt did not match the reality. It contributed to patchy implementation and sometimes fragmentation, inadequate considerations of the resources for implementation, and the opinions and experiences of frontline health workers needed to inform policy design. It was observed that faced with inadequate resources frontline workers preferentially invested their time in implementing well funded DP programs or activities that raised out of pocket fees from clients rather than comprehensive and integrated national policies and plans. Concern was also expressed that short term pilot projects deprived countries of the incremental knowledge to be gained from longer term implementation processes.

In Sierra Leone, one of the criticisms mentioned in the 2017 National Health Sector Strategic Plan (NHSSP) was that the plethora of policies developed around health care post-Ebola, while welcome, suffered from some level of fragmentation. Policies were developed in silo, without the input of a wide variety of stakeholders and in some cases, without attempts to synergize them with pre-existing policies. This has resulted in laws and policies that do not necessarily always align; and created gaps as well as overlap in terms of responsibility for both delivery as well as coordination of deliverables [42].

#### Capacity for inter and cross sectoral collaboration

*‘ … my big question is where is the Ministry of Finance … , where is the Ministry of Roads … , where is the ministry responsible for water ...Where is the Ministry of Education … ?’ (Development Partner Ghana).*

*‘we tried to involve other ministries and organizations. But we soon realized that whenever we called a meeting, other ministries and organizations sent people who could not commit their organizations to this thing … and that has been the failure of collaboration in Ghana’(MOH / State Ghana).*

Several KI felt that HP is integral to all three agendas and requires the ability to work with sectors beyond health. However effective intersectoral collaboration remains a challenge. Cross-sectoral coordination can be messy, and subject to political calculations, creating obstacles to effective cross-sectoral links.

Nearly, if not all, of the multiple plans and policies developed to respond to health shocks and epidemics post-Ebola in Sierra Leone incorporate multisectoral collaboration with representatives from partners, ministries and institutions outside of health. However, it was observed that the actual response is overly militarized, and anecdotally, there has been some reports about jockeying for power and influence between and among response agencies, as well as politicization of key posts and response efforts more generally.

#### Trust and accountability

*‘You can’t micromanage everything in life … there should be a little trust otherwise we can’t live’ (MOH / State Ghana).*

Trust and accountability between institutions, individuals and public and private sectors were seen as drivers of synergies if high and fragmentation if low. At the time of the study trust was unfortunately seen as low in both countries. The mistrust was not only in donor-government relationships but also between country institutions and system levels. In Ghana it was mentioned that sometimes DP did not trust the government and its agencies and vice versa. The DP mistrust was sometimes founded on accountability concerns.

*‘ …. If you have the systems in place, then the donor can say take my money because you can account for it. I only earmark if you can’t account for my money’ (Development Partner Ghana).*

In Sierra Leone despite calls for change, many programs implemented by DP and NGO continue to be vertical in nature [43]. A lack of trust in the political system as well as perceived weaknesses in state-run accountability systems mean that DP are still loath to use government institutions or processes and feel safer establishing parallel structures such as their own supply chain for drug procurement [41]. One way in which Sierra Leone has tried to minimize fragmentation and amplify synergies related to lack of accountability has been through the government requirement that DP sign Service Level Agreements (SLA) prior to implementing any projects. SLA are supposed to operate at all levels of government to ensure compliance and alignment with national health policies and avoid duplication of efforts and resources. In practice SLA are not routinely signed, and government finds it difficult to implement and monitor them.

### Country health systems

#### Leadership, institutional governance arrangements and structure

*‘I wouldn’t say it is inevitable. It can be changed. And it requires the ministry as a leader to be able to bring all the various parts to see that “this is the goal and these are the synergies that I am focusing on”’ (Non State Partner Ghana)*

*‘I don’t think fragmentation is inevitable. I think fragmentation is a creation of lack of policy direction and leadership’ (Development Partner Ghana)*

Country level exercise of agency through strong leadership came up consistently as a way of reducing fragmentation and increasing synergies. Several KI in Ghana felt fragmentation was not inevitable. However, it would continue to exist where there were leadership weaknesses combined with resource limitations. An example of leadership weaknesses causing institutional fragmentation that was brought up by many KI in Ghana was the leadership and management of the move from a unitary MOH to an implementation agency model following the passage of Act 525 of 1995 [44].

*“I think that the biggest fragmentation in Ghana was the GHS MOH split … .the crème de la crème moved to GHS leaving a weak MOH” (Development Partner Ghana)*

Act 525 created a health system organizational design where the MOH became a national level Civil Service institution responsible for coordinating policy planning monitoring and evaluation across larger service delivery and other agencies such as the Ghana Health Service (GHS) and the Teaching hospitals. KI felt such complex organizational reform needed leadership and a capacity to administer that was and remained missing.

*‘..the Act was not properly implemented and it’s still not being implemented … .in that Act the ministry is not supposed to do any implementation … but the ministry has jumped into implementation … they are in (implementation) simply because I understand that’s where the money is … ’(MOH /State Ghana).*

The long drawn covert as well as overt bureaucratic infighting and power struggles between MOH and its major service delivery agency GHS, over turf, power, position and control of resources was seen as having weakened and fragmented the health system. Successive waves of legislation that have created more implementing agencies, services and authorities – sometimes in reaction to strong pressure and lobbying by interest groups and individuals was felt to have worsened the fragmentation.

*‘you are having so many agencies, currently we have 26 agencies. The more you have these agencies, then the more fragmented you will become because the agencies are given different functions, and it makes them more fragmented … ’(MOH /State Ghana).*

Fragmentation driven by weak institutional arrangements and structure in the health sector and leadership also emerged in Sierra Leone. A winner -take-all political system where a change in party might mean a change also in key personnel with a loss of institutional memory, knowledge, skills and experience, was seen as a driver of fragmentation. One of the concerns expressed by some KI was that the current ruling party, was not in power during the Ebola outbreak. Only a handful of those who were involved in Ebola were now available to advice as many people affiliated with the previous administration had been let go. The result was an inability to sufficiently leverage the expertise and learning that was developed during the Ebola response.

#### Resources

*"[it] it boils down to the power of resources in the hands of donors and the often weakness of government to say no to funding for interventions outside the strategic framework of government," ( … … )." (MOH /State Sierra Leone)*

Health financing arrangements were described as a driver of fragmentation in both countries for several reasons. First was the inability to generate and also to commit sufficient financing for health on the part of government. Secondly, what funds flowed into the sector were unpredictable and slow. Thirdly funding was through diverse means and fragmented funding pools. Sometimes resources for some plans came from outside the responsible agency, necessitating time consuming discussions with other institutions and difficulties in accessing the money. Fourthly, the priorities of the DP who sometimes stepped in to fill the funding gaps, rather than national plans were droving implementation.

*"Donors tag their support to health priorities that fall within their strategic framework. The question often asked is whose reality counts?" (MOH /State Sierra Leone)*

However, the government was also blamed for contributing to the fragmentation by being more interested in receiving donor funds than in ensuring donor alignment with government agendas and national priorities.

*"Fragmentations are inevitable in the sense that donors provide the much-needed resources and the tendency is that we go for the windfall. Example, the Global Fund was down loaded on us. We went for the money regardless of whether it reflects a priority health need in our country" (MOH /State Sierra Leone ).*

Internal as well as external funding flows were mentioned as contributing to fragmentation in Ghana. For example, the NHIS fund supports clinical care mainly and ignores health promotion. Additionally, the fund is inadequate for the services guaranteed free under the policy and delays in provider reimbursement has led to the overt and covert withdrawal of certain services by providers.

Some KI in Ghana felt some fragmentation was inevitable because of resource constraints and the need to prioritize.

*‘ … because of limited resource envelopes, you cannot build a system in its holistic nature. No matter how rich a country is. So, you need to prioritize pathways to achieve priority goals … ’. (Development Partner Ghana).*

*‘ … , we cannot do away with it, you can’t’ because it boils down to funding. … (Non State Partner Ghana).*

*‘fragmentation is part of human nature. I mean even in your own house there may be some divisions, but we can manage it’ (MOH /State Ghana).*

#### Health information systems (HIS)

By providing information on morbidity, mortality and health system performance, a well-functioning health information system can contribute to synergies between UHC, HS and HP. However, for this to happen there needs to be capacity within the health sector to manage and utilize the information. One of the focal areas of the policies developed post-Ebola was to strengthen Sierra Leone’s health information systems (HIS). HIS weaknesses contributed to the initial inability to detect the scope of the EVD outbreak.

## Discussion

Concerns about fragmentation in agenda setting, formulation and implementation of global health agendas in low and middle income countries (LIMC); and expressed desire for more synergies or even integration are not new. Multiple global level efforts to promote synergies and reduce fragmentation, often initiated and led by global multi-lateral agencies and wealthier countries that provide development assistance to LMIC have emerged over time and continue to emerge [45–49]. At the global level, several inter-related problems or challenges account for the persisting difficult in reducing fragmentation and increasing synergies. They include the proliferation of global health actors, global health leadership, accountability, power relations and divergent interests [50] Focusing efforts to reduce fragmentation and increase synergies predominantly at the global level is clearly challenging. Is the situation any different at country level in LMIC? In this study we have tried to understand how and why fragmentation and synergies occur (or not) in policy and program agenda setting, formulation and implementation at country level in Ghana and Sierra Leone between the global health agendas of UHC, HS and HP.

A normative assumption underlying our starting position as researchers was that whenever possible, synergistic approaches to global health agenda policy and program agenda setting, formulation and implementation are better than fragmented approaches if low and middle income countries are to sustainably, effectively and efficiently implement global health agendas to improve population health. We did not try to empirically test this normative assumption. Rather we focused on trying to understand how and why synergies and fragmentation occur or not. We have identified multiple factors (Fig. 1) that pull towards fragmentation or push towards synergies.

The drivers we found include some well know challenges related to global actors and institutions [50] However we also found country level drivers such as the nature of the policy process, country context, how actors exercise agency and power as well as health systems leadership and structural factors. Despite global power imbalances often related to resources and technical expertise, our study suggests countries like Ghana and Sierra Leone have agency and power to influence synergies and fragmentation. The problem is perhaps more of how well (or not) this potential is recognized, developed and mobilized. Mobilization of country level actor agency and power requires political and bureaucratic commitment to and prioritization of synergies, technical expertise and skilled leadership to counter sometimes daunting contextual, system and structural constraints.

The stated shared goal among different global and country health system actors and stakeholder to see improved population health outcomes, does not necessarily translate into shared objectives and targets as to how to attain this goal. Actors who push for and support fragmented approaches as well as those who support synergistic approaches all argue that their approach is the best way to attain population health outcome improvement in the face of resource constraints. The shared interest in population health outcome improvement needs to be actively harnessed by country level actors to bring different actors together to influence definition and implementation of a clearly articulated vision and ideology of a shared approach to attaining population health outcome improvement that actively looks for and implements useful synergies. The Ghana SWAp of the 90’s that several of our respondents described did this until its gradual demise related to global and national leadership actor transitions and ideology shifts.

Another shared interest, that nevertheless translates into different approaches is the need for global as well as country actors to demonstrate results and have visibility of effort. The divergence is related to the fact that the constituents to whom the results of efforts to improve population health need to be made visible are not necessarily be the same. In LMIC multi-party democracies like Ghana and Sierra Leone, to remain in power from one election to the next; governments need to demonstrate valued results to citizenry who live day to day in that context and ‘next door’ to decision makers. Citizens are more likely to be impressed by efforts that improve the totality of their day to day lives. Such efforts often have to be multi-agenda, multi-sectoral and synergistic. Development partner agencies on the other hand need to demonstrate valued results to governments and citizens in countries somewhat remote from the day-to-day context and lives in the LMIC they are supporting. A brochure or documentary showing dramatic improvements in selected measurable dimension such as immunization coverage or access to anti-retrovirals is relatively quickly achievable and will impress. The implication of this is that without collaborative mechanisms and skills that enable dialogue and arrival at common agreed outputs and how to share visibility; fragmentation is likely to persist as different actors try to maximize their interests.

We also found that accountability and trust (or the lack thereof) within health systems influence fragmentation and synergies. Accountability refers to the “obligation of individuals or agencies to provide information about, and / or justification for their actions to other actor along with sanctions for failure to comply” [51]; and trust to “the optimistic acceptance of a vulnerable situation in which the trustor believes the trustee will care for the trustors interests” [52] Trust underpins cooperation within health systems but remains an under-researched area in low and middle income country health systems [53, 54]. For actors to be willing to consider synergistic approaches, attention needs to be paid to understanding and building trust and accountability within health systems. This must include clear agreements on obligations and commitments as well as enforcement mechanisms and structures that promote and maintain trust and accountability.

The global as well as the national context in which national health systems function affects decision making towards. Context is hard to control and contextual change requires long term effort in many sectors. A more practical approach to achieve short and medium as well as long term results, despite contextual constraints; is for health systems to learn how to change to get round the limitations of context, maneuver within context and mobilize their strengths to synergistically achieve goals and targets.

Finally, country level policy and program actors as well as citizens perhaps have to gain an increased understanding of power and how to better mobilize it to support achievement of population health improvement. For example, in both countries, we heard the narrative from KI that DP domination of health financing at country level is a major driver of continuing fragmentation. However, the Sierra Leone National Health Accounts (NHA) for 2013 shows that out of pocket payments by citizens of Sierra Leone accounted for 62% of Total Health Expenditure (THE). This was higher than the percentage from Government of Sierra Leone general taxes (7%) or DP (24%) [55]. Similarly the Ghana 2015 NHA data shows that 75% of health sector financing was domestic while 25% was from abroad [56]. Out of pocket payments are inequitable; but that does not change the fact that the narrative that arises from examining the NHA data in both countries should be that citizens are the major financiers of the health system. If major financiers are what drives fragmentation or synergies, where are the citizen voices? This is not to downplay the importance of DP contributions in LMIC; but to draw attention to narratives that can end up exerting power as thought control, marginalize some key stakeholders and block effective negotiation of priorities.

Experienced, competent, knowledgeable and committed health sector leadership as well as appropriate technical analysts [57] are essential to help Ministries of Health lead effectively to prioritize synergies and minimize harmful fragmentation. Health sector leadership in LIMC needs to mobilize agency to pull towards synergies and push back against fragmentation with vision, technical skills and credibility as well as political and bureaucratic prioritization of synergies promotion.

## Conclusions

Synergies and fragmentation in policy and program agenda setting, formulation and implementation for the global health agendas of UHC, HS and HP occur in both our study countries.

Fragmentation was generally perceived as disruptive, even though a few actors argued that fragmentation is sometimes needed to achieve population health improvement goals rapidly. Synergies and fragmentation are driven by multiple factors including country and global level power imbalances. Country actors have agency and power that can be mobilized to tilt the balance of power away from fragmentation and towards synergies. This requires country level awakening to the latent potential, as well as political and bureaucratic commitment to and prioritization of beneficial synergies. It also requires skilled and disinterested country technical expertise and leadership at all levels to counter the daunting contextual, system and structural constraints to more synergistic approaches to framing and implementing global health agendas in low and middle income countries like Ghana and Sierra Leone.

### Study limitations

This was a qualitative case study of two countries. The theory generated needs to be further tested with more country case studies – the equivalent of replication in experimental studies – to confirm its generalizability or otherwise.

## Supplementary Information

**Additional file 1.** Key Informant Interview Topic Guide

## Data Availability

The anonymized interview notes and transcripts are available from Irene Akua Agyepong, the country PI in Ghana (iagyepong@gcps.edu.gh) and Fredline M’Cormack-Hale the country PI in Sierra Leone (fredline.mcormack-hale@shu.edu). The KI interview guide is made available as a supplementary file.
