# Supplementary material for: Synergies and fragmentation in country level policy and program agenda setting, formulation and implementation for Global Health agendas: a case study of health security, universal health coverage, and health promotion in Ghana and Sierra Leone
Source: BMC Health Serv Res. 2021 May 20;21:476. doi: 10.1186/s12913-021-06500-6 (PMC8134803; doi:10.1186/s12913-021-06500-6)
Supplement: Supplementary file 1 — Additional file 1. Key Informant Interview Topic Guide [file 12913_2021_6500_MOESM1_ESM.docx]

Synergies and Fragmentation in country level policy and program agenda setting, formulation and implementation for Global Health Agendas: A Case Study of Health Security, Universal Health Coverage, and Health Promotion in Ghana and Sierra Leone

Authors

Irene Akua Agyepong (Corresponding author)

Ghana College of Physicians and Surgeons, 54 Independence Avenue, Accra / Dodowa Health Research Center, P.O. Box DD1, Dodowa

Email: [iagyepong@hotmail.com](mailto:iagyepong@hotmail.com) /iagyepong@gcps.edu.gh

Fredline A. O. M’Cormack-Hale
Associate Professor, School of Diplomacy and International Relations
109 McQuaid Hall, Seton Hall University, 400 South Orange Avenue
South Orange, NJ 07079
Phone: (973) 313-6065

Email: fredline.mcormack-hale@shu.edu

Hannah Brown-Amoakoh

Noguchi memorial Institute for Medical Research, University of Ghana

P.O. Box LG 581, Accra, Ghana

[hamoakoh@noguchi.ug.edu.gh](mailto:hamoakoh@noguchi.ug.edu.gh%20%20) /[h.b.amoakoh-2@umcutrecht.nl](mailto:h.b.amoakoh-2@umcutrecht.nl)

University Medical Center, Utrecht University

P.O Box 855003508 GA Utrecht, The Netherlands

Abigail **N. C.** Derkyi-Kwarteng

Ghana Health Service / Ghana College of Physicians and Surgeons

Email: **nyarkocodjoe@yahoo.co.uk**

Theresa Ethel Darkwa

Public Health Division

37 Military Hospital

Neghelli Barracks

Cantonments, Accra

Email: [teri9664@yahoo.com](mailto:teri9664@yahoo.com)

Wallace Odiko-Ollennu

Non-Communicable Diseases Control Program

Ghana Health Service

P.O.Box KB 493

Korlebu, Accra

email: odikobi@yahoo.com

Mobile: 0548228599

## Key Informant Interview Topic Guide

1. Who are the main actors in UHC, GHS and HP policy agenda setting, formulation and implementation in Ghana /Sierra Leone?
2. Which ministries are relevant in Ghana /Sierra Leone with regards to UHC, GHS and HP policy agenda setting, formulation and implementation?
3. In any of the ministries, are there particular units spearheading UHC/HP/GHS in Ghana /Sierra Leone? (or any other government/quasi-government bodies involved in/responsible for UHC/HP/GHS)
4. What are the agendas, interests, priorities, power (political and financial) of these actors?
5. Which actors pursue a ’complete’ agenda, which actors pursue an ‘incomplete’ agenda (UHC, GHS, HP, or elements thereof)?
6. Who are the main donors in your country?
   ESPECIALLY/PROBE on ( WHO; GFATM; WORLD BANK (+GFF); GAVI;. UNDP; UNICEF: GATES; USA (+PEPFAR) (+GHSA?); UK; GERMANY; FRANCE; JAPAN) (Donor Presence, Vertical programme coordination bodies* (includes government-led) donor/development partner coordination bodies as well as those for coordination across gov’t departments)
7. What are their agendas/priorities?
8. Is UHC/HP/HS (or any of them) supported/not supported by influential politicians or the political climate of your country? CONSIDER Office of the PM / President as well as Ministers, Legislature /Parliament etc.*
9. Are there any coordinating bodies for either UHC, HP or HS in your country?
10. CSOs presence CSOs influence: Who are the main CSOs (or consortia of CSOs) in your country?
    What are their agendas/priorities?
11. Are there particular CSOs (or consortia of CSOs) spearheading UHC/HP/GHS in your country?
12. Is there media and/or professional association involvement /spearheading of UHC/HP /GHS in Ghana /Sierra Leone
13. In your country is there an alignment between political structure and donor activities (including money flow)? Are there particular donors spearheading UHC/HP/GHS in your country?
14. Are communities in your country responding or engaging in addressing UHC/HP/HS in your country? If yes, how?
15. Is there a coordination/cooperation between governments and CSOs (e.g. meetings to discuss and agree on mutual interests)?
16. Who are the main private sector organizations/institutionalized structures in your country? (e.g. trade unions, churches, …). Private for profit healthcare providers; Private non-profit healthcare providers
17. What are their agendas/priorities?
18. Are there particular private sector organizations/institutionalized structures spearheading UHC/HP/GHS in your country?
19. How much money do these private sector organizations/institutionalized structures give or get in relations to UHC/HP/HS in your country?
20. Is there a coordination/cooperation between governments and private sector organizations/institutionalized structures (e.g. meetings to discuss and agree on mutual interests)?
21. In your country is there an alignment between political structure /will and private sector organizations/institutionalized structures activities?
22. Who are the main academic persons/organizations/institutionalized structures in your country involved in UHC/HS/HP?
23. What are their agendas/priorities?
24. Are there particular academic persons/organizations/institutionalized structures spearheading UHC/HP/HS in your country?
25. Is there a coordination/cooperation between governments and academia (e.g. meetings to inform, discuss and agree on mutual interests)?
26. Are there any training or research programmes for UHC/HP/HS in your country?
27. Who is entitled to these training programs? How often are they provided?
28. What do they cost? Are there funds for training or research for UHC/HP/HS in your country?
29. What are people's experiences of this fragmentation?
30. Regarding the above discussed, do you see any areas of unrealised synergies regarding UHC/HS/HP?

## Supplementary Questions to help further exploration (As relevant)

1. What, in your opinion, are major forms of fragmentation of health systems, and how do they pertain to the three agendas?
   (Aspects: institutional, financial, etc.)
2. Do you (as an actor in the Ghana /Sierra Leone health system) do you think you contribute in any way to these/some of these fragmentations? How/In which way?
3. Are these fragmentations inevitable? If yes, why? If no, what could or should be changed?
4. Do you see potential synergies with regards to UHC/HS/HP?
5. Are you (as an actor in the Ghana /Sierra Leone health system) engaged in/seek synergies? If yes, how?
6. If you are not seeking synergies with other efforts, why not, why?
   (e.g. Limited acknowledgement of complexity of health systems, desire or pressure to achieve rapid results, considering fragmentation as relatively unproblematic, underestimating potential synergies, ...)
